# Burnout Syndrome and its associated factors among anesthesia technicians in a Teaching Hospital in the central region of Tunisia

**DOI:** 10.1192/j.eurpsy.2023.992

**Published:** 2023-07-19

**Authors:** Z. Athimni, A. Chouchen, H. Kalboussi, L. Nsiri, F. Ferhi, A. Aloui, A. Gaddour, M. Bouhoula, M. Maoua, A. Brahem, O. Maalel, S. Chatti, I. Kacem, N. Mrizak

**Affiliations:** 1 Service de médecine du travail et de pathologies professionnelles - CHU Farhat Hached 4002 Sousse, Tunisie; 2 Service d’anesthésie réanimation CHU Farhat Hached 4002 Sousse, Tunisie; 3 Service de médecine du travail, Hôpital Régional Ibn El Jazzar Kairouan, Tunisi, Université de sousse, Sousse, Tunisia

## Abstract

**Introduction:**

Burnout (BO) is a syndrome combining psychological and somatic symptoms caused by exposure to several years of chronic stress at work. Far from being a theoretical problem, it is a real social problem that has become globalized as societies change. Anesthesiology is among the most stressful medical disciplines which expose to BO in Tunisia and around the world.

**Objectives:**

Identify associated factors to BO among anesthesia technicians.

**Methods:**

We conducted a cross-sectional study during two months, from October 1^st^, 2015 to December 31^th^, 2015, among anesthesia technicians affected in the different operating rooms of the Farhat Hached teaching Hospital in Sousse. Data collection was based on a self-administered questionnaire with validated tools assessing BO (Maslach Burnout Inventory) and stress (Siegrist and Karasek)..

**Results:**

Forty-six senior anaesthesia technicians was included in the study. The mean age of our population was 43.76 ±7.74 years with a female predominance (89.1%). According to the Karasek model, 59% of the workers were in job strain, and according to the Siegrist model 23.9% of the participants had an imbalance between high effort and low reward. The BO rate among anaesthesia technicians at the Farhat Hached University Hospital was 39.1%. The results showed a statistically significant association between working at the gynaecology-obstetrics department (p=0.001), the seniority in the department superior or equal to 20 years (p=0.006), the absence of break time at work (p=0.003) and the risk of the occurrence of BO. Furthermore, the last 2 consecutive day rest dates back to more than 15 days (p=0.001), the number of free weekends during the last 3 months less than four (p=0.044) were also significant associated to BO.

**Conclusions:**

Our study confirms that BO is a tangible reality in our country especially among anesthesia technicians, so it must be addressed by adopting effective preventive strategies.

**Disclosure of Interest:**

None Declared

